# Reproductive Traits of an Invasive Alien Population of Grey Squirrel (*Sciurus carolinensis*) in Central Italy

**DOI:** 10.3390/ani10040738

**Published:** 2020-04-24

**Authors:** Margherita Maranesi, Antonello Bufalari, Cecilia Dall’Aglio, Daniele Paoloni, Giulia Moretti, Silvia Crotti, Elisabetta Manuali, Marica Stazi, Francesca Bergamasco, Deborah Cruciani, Antonio Di Meo, Cristiano Boiti, Massimo Zerani, Francesca Mercati

**Affiliations:** 1 Department of Veterinary Medicine, University of Perugia, via San Costanzo 4, 06126 Perugia PG, Italy; margherita.maranesi@unipg.it (M.M.); giuliamoretti89@gmail.com (G.M.); francescaberga95@gmail.com (F.B.); antonio.dimeo@unipg.it (A.D.M.); boiti.cristiano@gmail.com (C.B.); massimo.zerani@unipg.it (M.Z.); francesca.mercati@unipg.it (F.M.); 2 OIKOS Institute, Via Crescenzago 1, 20134 Milano MI, Italy; daniele.paoloni@istituto-oikos.org; 3 Experimental Zooprophylactic Institute of Umbria and Marche “Togo Rosati”, via Gaetano Salvemini, 1, 06126 Perugia PG, Italy; s.crotti@izsum.it (S.C.); e.manuali@izsum.it (E.M.); m.stazi@izsum.it (M.S.); d.cruciani@izsum.it (D.C.)

**Keywords:** alien invasive species, uterine scar, morphometry, grey squirrel, reproduction, red squirrel, competitive exclusion

## Abstract

**Simple Summary:**

The presence of an invasive alien species is one of the main causes of biodiversity loss, which occurs through the reduction or even the extinction of native populations. In this context, the EU 2020 biodiversity strategy calls for research on invasive alien species in order to prevent their introduction and spread more effectively. The grey squirrel (*Sciurus carolinensis*), which is native to North America, provides one of the best-known examples of competitive exclusion of a native one, the European red squirrel (*Sciurus vulgaris*). A grey squirrel population, living in central Italy (Umbria), was studied to provide parameters on its population dynamics, growth rates, and viability, in order to understand its adaptation to its non-native range. Our data revealed that the Umbrian grey squirrel population is well-adapted to its new range, undergoing two annual mating periods and experiencing high reproductive success. Therefore, these invasive grey squirrels pose a substantial threat to the local European red squirrel population.

**Abstract:**

The reproductive cycle of an invasive alien Italian grey squirrel population was studied to understand its adaptation and limit its spread, in order to conserve the autochthonous red squirrel. Female and male genital traits were evaluated throughout the reproductive cycle, including the ovary, uterus, testicle, epididymis, seminiferous tubule morphometry, and germinative epithelium histology. Moreover, individual female fecundity was determined by counting uterine scars. Ovary width and uterus weight, length, and width reached their highest values in the luteal and pregnancy phases. On conducting a histological evaluation of the testicular germinal epithelium, four morphotypes related to the different reproductive phases of the male squirrels were identified: immature, pubertal, spermatogenesis, and regressive. Testicle and epididymis weights and seminiferous tubule diameters reached their largest values during spermatogenesis. Uterine scar analysis showed that 69% of the females had given birth to one or two litters, while 31% had no uterine scars. Litters were larger in the first breeding period than in the second; annual fecundity was 4.52 ± 1.88 uterine scars/female. Umbrian grey squirrels have adapted to their non-native range, showing two annual mating periods at times similar to those in their native range, and high reproductive success.

## 1. Introduction

Invasive alien species (IAS) are one of the major drivers of biodiversity loss [1]. These IAS can reduce or promote the extinction of native populations worldwide through different ecological processes [2], including interspecific competition [3], predation [4], transmission of infectious diseases [5], passing on helminths causing parasitological illness [6], and changes in ecosystems [7]. 

One of the aims of the EU 2020 biodiversity strategy is to study IAS. In particular, the EU Regulation 1143/2014, on the prevention and management of the introduction and spread of IAS, makes this recommendation. One of the best-known examples of competitive exclusion of a native species by an alien species is the replacement of the Eurasian red squirrel (*Sciurus vulgaris*) by the Eastern grey squirrel (*Sciurus carolinensis*) in Great Britain, Ireland, and Italy [8,9,10]. 

The grey squirrel belongs to the order of Rodentia, family Sciuridae, and is native to eastern North America; this medium-sized squirrel lives mainly in trees and has no sexual dimorphism, neither in body size nor in coat colour [11]. The grey squirrel is one of the most invasive species in Europe and worldwide; accordingly, it has been included among the 100 World’s Worst IAS by the International Union for the Conservation of Nature [12]. Consequently, trading and breeding of the grey squirrel is currently prohibited, even if they are still imported and traded illegally in several countries [13]. The grey squirrel initially expanded its range in several states of the USA and Canada [11]; from there, it was introduced to South Africa [11], Great Britain [8], and Italy [10]. Eradication and sterilization studies have recently been carried out in several Italian regions, including Piedmont [14], Lombardy [10,15], and Umbria [16]. The dynamics of how the initial invasion of grey squirrels took place in Umbria remain unclear. Seven grey squirrels were purchased in 1999 and placed in large cages inside a zoological garden near Perugia [16]. The squirrels managed to escape from their cages in the early 2000s [16], and expanded their range over at least 50 km^2^, in a natural area close to Perugia connected to nearby Apennines woodlands. The LIFE BIO/IT204 U-SAVEREDS Project was set up for the conservation of the European red squirrel in Umbria [17], at least in part owing to the threat imposed by invasive grey squirrels.

The negative interaction between the two species has been attributed to competition for food resources and habitats, thus reducing breeding and recruitment [3,18], but it also due to the chronic stress induced by co-existing in the same habitat [19], and to interspecific competition leading to behavioural changes [20].

Eastern grey squirrels can have two breeding seasons; generally, most breeding occurs in December–February and May–June [11,21,22]. Consequently, after 44 days of gestation [21,23,24], the females deliver a spring litter (or first litter) and/or a summer litter (or second litter) [21]. The lactation period may last as much as 70 days [23]; the young are weaned at 8–10 weeks old [21], and sexual maturity occurs at about 10–12 months [21].

In male grey squirrels, spermatogenesis generally peaks during the female breeding season [11,21]. Nevertheless, male squirrels undergo periods of sexual regression and redevelopment [25]. There is disagreement as to whether the regression is synchronous throughout the population, or if it is an individual phenomenon. In the same country, Britain, both phenomena were described: sexually regressed males were found during all months of the year [26], but a seasonal cycle of sexual regression, in the autumn, and redevelopment was also described [25]. Similar findings were described in USA [27], but with a greater synchronization of the male reproductive cycle [21,28].

The growth rate of a population is affected by annual fecundity: the product of the number of weaned young per litter and the number of litters produced per year by females [3]. Fecundity rates can be estimated by counting the number of embryos and uterine scars if carcasses of females are available [29,30,31]. Uterine scars form at embryo implantation sites in the utero and can last up to nine months after parturition [29,30,31]. Two successive pregnancies can be also identified because uterine scars have a different colour intensity [15,32]. 

Studies of the reproductive cycle of an IAS can establish parameters on population dynamics, growth rates, viability, and ultimately allow more precise estimates of how quickly populations of invasive species will spread in their non-native range. These demographic parameters can predict the management effort required (i.e., number of animals to be culled) to achieve population reduction successfully or to estimate future spread rates and demographic trends in population dynamics models [33,34]. Bearing this in mind, the reproductive cycle and fecundity of the Umbrian grey squirrel population was studied by investigating uterine scar evaluation and litter production through the analysis of female and male genital tract morphometric and morphological seasonal changes.

## 2. Materials and Methods 

### 2.1. Animal Capture and Sample Collection

Sixty-three female and 51 male grey squirrels were captured using Tomahawk live traps (Model 202.5, Tomahawk Live Trap Co., WI, USA) during the 2016–2018 control campaign. The traps, baited with hazelnuts, were placed 30 to 100 m away from sunlight or busy roads to limit, as far as possible, additional stress on captured squirrels [16]. Each trapping session lasted for a minimum duration of 10 days. Trapping was sometimes carried out for several consecutive months until the bait was entirely consumed. The traps were normally checked twice a day in the middle of the morning and before sunset. None of the squirrels died inside the trap or suffered fatal injuries. The squirrels were captured in compliance with regulations regarding wildlife control laid down in Article 19 of the Italian Law 157/92: “Rules for the protection of wild animals and homeotherms and for hunting”.

Body weight, sex, and reproductive morphological characteristics, including testicle positions (abdominal versus scrotal), as well as scrotum (scrotal colour, hair coverage, and staining) [35] and vulva (colour and dimension) [11] features were recorded for each captured squirrel in order to compare their estimated age and sexual maturity with the morphological and histological traits of their genital organs. Forty-five females and 18 males were subsequently euthanized by CO_2_ inhalation, following EC and AVMA guidelines [36,37,38], while 18 females and 33 males were surgically gonadectomised [39] in order to collect ovaries, uteri, testicles, and epididymides.

### 2.2. Morphometric Evaluation of the Female Genital System and Uterine Scar Analysis 

The female reproductive apparatus, from ovaries to vagina, was collected and stored in tap water at −20 °C up to two months [15]. After thawing at room temperature, the female reproductive apparatus was analysed and the following information was acquired: weight, length, and width of each ovary; presence of mature follicles and corpora lutea; uterus morphology and weight; length and width of the uterus at the level of both uterine horns (measured between their proximal extremities); and length and width of the medial distal region of the uteri. A vernier calliper and analytical balance were used to measure the samples. The length of the uterine horns was measured after cutting the broad ligament and stretching them to their maximum extent.

Evaluation of uterine scars was performed as previously described [15]. In short, the uterus was opened with a longitudinal cut in order to expose the endometrium and was then immersed in a fresh solution of 10% ammonium sulphide for 10 min. After washing in tap water, each uterine sample was incubated for 10 min in a fresh solution of 1% hydrochloric acid and 20% potassium hexacyanoferrate mixed together in equal parts just before use. After washing in tap water, the uterine scars were counted within minutes using a dissecting microscope in order to prevent changes in colour over time. Scars were considered to be recent or old based on the colour intensity of their bands [15,16]. All the information acquired from the study of the genital apparatus was used to estimate the reproductive phases of the female squirrel.

### 2.3. Morphometric Evaluation of the Testicles

Immediately after taking the testicles and epididymis samples, the following data were recorded: testicle and epididymis weight, as well as testicle length and width. A vernier calliper and analytical balance were used to measure samples. The testicles were then immediately fixed in a 10% formaldehyde solution in phosphate-buffered saline (PBS, 0.1 M, pH 7.4) and processed before embedding them in paraffin wax. Five-micrometres-thick sections, mounted on poly-L-lysine-coated glass slides and air dried at 37 °C, were stained with Haematoxylin–Eosin [40]. Germinal epithelium and the diameter of seminiferous tubules were evaluated histologically. Ten tubules for each sample were measured using the photomicroscope (Nikon Eclipse E800, Nikon Corp., Tokyo, Japan) connected to a digital camera (Nikon Dxm 1200 digital camera) [41]. Histological sections were analysed at 20X magnification and measured using image analysis software (Lucia Measurement, Laboratory Imaging Ltd, Praga, Czech Republic). All the information acquired from the study of the genital apparatus was used to estimate the reproductive phases of the male squirrels.

### 2.4. Statistical Analysis

Morphological variations have been compared to the female and male reproductive phases (Table 1 and Table 3, respectively). All morphological data (weight, length, width, diameter, and uterine scar number) were analysed by Levene’s test and by one-way ANOVA followed by a Duncan–Newman–Keuls t-test. Differences were considered significant at *p* < 0.01.

## 3. Results

### 3.1. Morphometric Evaluation of the Female Genital System

**Figure 1:**
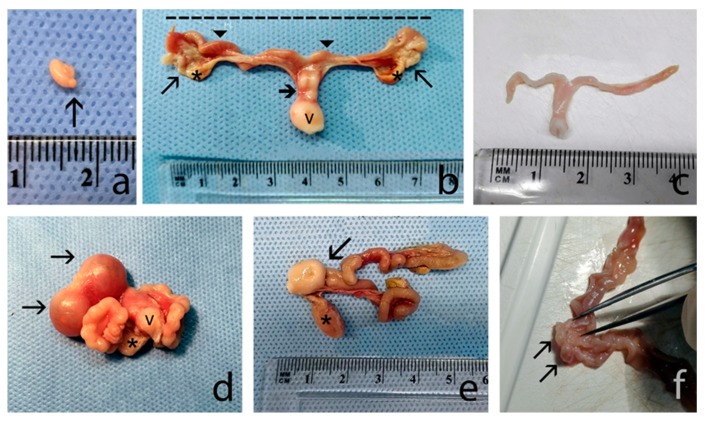
(**a**) An ovary with a corpus luteum (arrow) on the surface. (**b**) A reproductive apparatus, after cutting the broad ligament and stretching, composed of the ovaries and fallopian tubes (arrows), uterine horns (arrowhead), and vulva (v); two uterine horns placed side by side in their distal end (bold arrow). The broad ligament infiltrated by adipose bodies (*) can be observed. The dashed line indicates that the length of the uterus was measured between the two proximal ends. (**c**) A white, small, ribbon-like and quite relaxed uterus belonging to an immature squirrel. (**d**) Two embryonic vesicles (arrows) are contained within a uterus strongly folded back on itself and forming spiral loops. Bladder (*) and vulva (v) are shown. (**e**) Dorsal surface with a transverse groove (arrow) defining the border between the cervix and the vagina. The bladder (*) can be observed below the vagina. (**f**) Distal and medial portion of the two uterine horns opened to show longitudinal folds of the mucosa. The arrows indicate the two external orifices of the uterus.

Female body weight was higher (*p* < 0.01) during the luteal and pregnant phases compared to the immature one (Table 1). Ovaries were spherical or oval and completely contained within the ovarian bursa (Figure 1a). No differences were evident in ovary weight and length among the different reproductive phases, while ovarian width was significantly greater during the luteal and pregnant stages than during the other stages of the annual female reproductive cycle (*p* < 0.01; Table 1). The ovarian surfaces were either smooth or irregular due to the presence of follicles and corpora lutea (Figure 1a). The fallopian tubes were thin, convoluted, and close to the ovaries. The duplex uterus, which has two uterine horns and two cervical canals that flank the distal portion and open separately into the vagina (Figure 1b,f), showed differences in shape and size among the different groups. In some of the squirrels, it was small, ribbon-like and quite relaxed (Figure 1c), while in others, it was rather bulky, oedematous, and strongly folded back on itself, forming spiral loops (Figure 1d). The colour ranged from white or light pink, usually when it was small, to dark red. Intercornual ligaments were present, but the dorsal ligament appeared to be more developed. On the dorsal surface of the uterus, a transverse groove defined the border between the cervix and vagina (Figure 1e); however, the groove was not visible in small uteri likely belonging to young animals. The weight, length, and width of the uterine horn and body reached their maxima (*p* < 0.01; Table 1) during the luteal phase and pregnancy (Table 1). One or more embryonic vesicles and foetuses were found in the pregnant squirrels, which were located in a single uterine horn or in both (Figure 1d). The broad ligament was always present, but in some animals, it contained adipose bodies located near the ovaries (Figure 1b).

Combining the data collected allowed to discern four distinct phases of the squirrel’s reproductive cycle as described in Table 1. These included the immature, follicular, luteal, and anoestrus periods for females that were seasonally distributed, as shown in Table 2.

### 3.2. Uterine Scar Analysis

**Figure 2:**
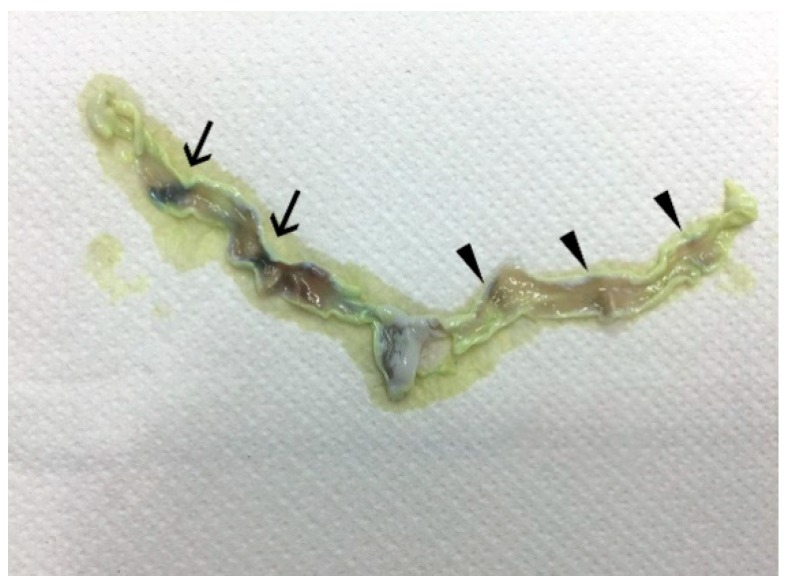
A uterus after the treatment to evidence uterine scars. The arrows highlight more recent darker scars, while the arrowheads indicate older, lighter-coloured scars.

The uterine scars appeared as two dark bands separated by an intermediate clear band. The most recent scars were dark, while the older scars were lighter in colour (Figure 2); both types of scars were found in the same uterus providing indications on the number of litters produced by the grey squirrel per year. Scars were never observed in the distal portion of the uterine horns; they were variably present in one or both uterine horns of sexually mature animals but not in those of immature animals (Table 1). Uterine scar analysis showed that 69% of the females had given birth to one (38%) or two litters (31%), while 31% had no scars. The litter size in the first breeding period was about twofold larger (*p* < 0.01) than the second breeding period (Table 3).

### 3.3. Morphometric and Histological Evaluation of the Testicles

**Figure 3:**
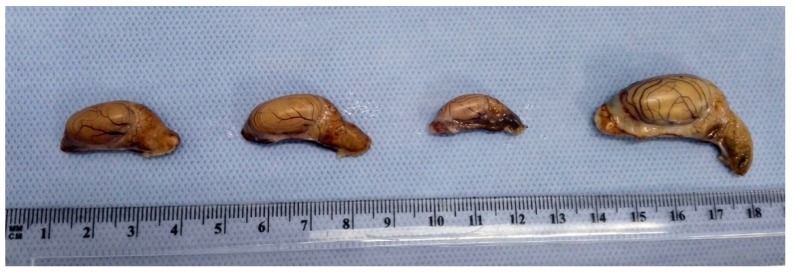
Testicles belonging to squirrels of different ages and breeding status.

Male body weight was higher (*p* < 0.01) during the pubertal, spermatogenesis, and regression phases than for immature males (Table 4). The testicles were oval and closely associated with the epididymides (Figure 3); their surfaces were smooth with clearly visible blood vessels below the albuginea tunic. A progressive increase (*p* < 0.01) in testicle and epididymis weight was recorded from the immature phase through to the spermatogenesis phase (Table 4). 

The testicle length was shortest (*p* < 0.01) in immature animals relative to other reproductive phases, while width was significantly greater (*p* < 0.01) during spermatogenesis than during the other phases (Table 4). The diameters of the seminiferous tubule reached their largest (*p* < 0.01) size during spermatogenesis (Table 4).

**Figure 4:**
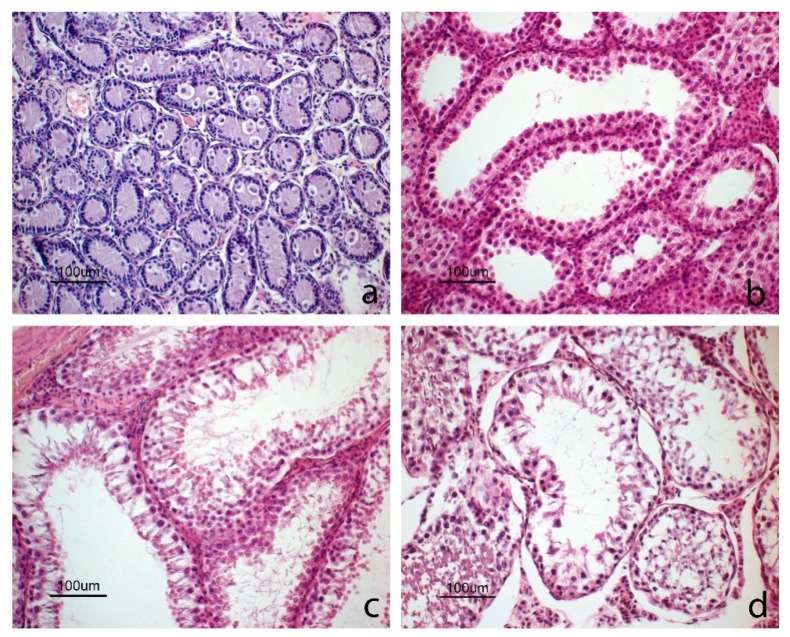
Images of testicles in different reproductive phases. (**a**) Immature phase: germinal epithelium where tubules are small and devoid of lumen; the epithelium consists of numerous Sertoli cells located at the base and some spermatogonia with abundant cytoplasm in a more central position. (**b**) Pubertal phase: some tubules have large lumen and the epithelium consists mainly of basal Sertoli cells and primary spermatocytes in the luminal compartment; round spermatids and a cluster of Leydig cells can also be observed. (**c**) Spermatogenesis phase: all tubules show a large lumen; epithelium is completely formed and is in active spermatogenesis with elongated spermatids. (**d**) Regression phase: testicles with degenerated germinal cells occupying the lumen.

Based on the histological evaluation of the germinal epithelium, which reflects breeding status, male squirrels were grouped into four morphotypes (Mp). The first group included squirrels with immature epithelium (Mp1; Figure 4a), characterized by small-diameter seminiferous tubules and devoid of lumen. The epithelium had numerous Sertoli cells located at the base and some spermatogonia in a more central position. The Leydig cells situated in the connective compartment, had large nuclei and little cytoplasm. These immature squirrels had low testicular weights (Table 4). 

**Figure 5:**
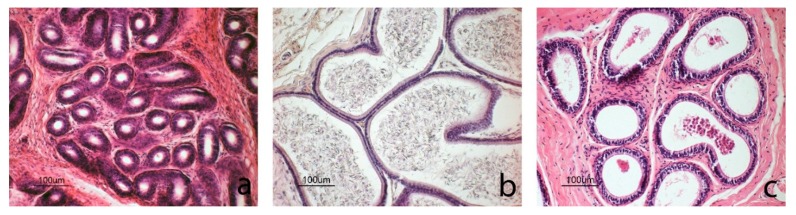
Images of epididymis in different reproductive phases. (**a**) Immature phase: the diameter of the epididymis is small and the lumen is empty. (**b**) Spermatogenesis phase: the diameter of the epididymis is larger and it is full of spermatozoa. (**c**) Regressive phase: cell debris and squamous cells can be observed in the epididymis lumen.

The second group was comprised of pubertal squirrels (Mp2; Figure 4b) with seminiferous tubules larger in diameter compared to the previous group (Table 4). Many tubules had a large lumen with epithelium composed of basal Sertoli cells and primary spermatocytes. In some tubules, the epithelium showed different cell types, including Sertoli cells, spermatogonia, primary spermatocytes, and round spermatids; however, no elongated spermatids nor spermatozoa were visible. In the first two groups, no spermatozoa were observed in the epididymis (Figure 5a).

The third group included sexually mature and active male squirrels (Mp3; Figure 4c) characterized by large tubules with lumen. The active germinative epithelium was composed of all spermatogenic cell types, including spermatids and spermatozoa. Leydig cells were clustered and had small nuclei and abundant cytoplasm. The epididymis was full of spermatozoa (Figure 5b).

The fourth group included individuals with testicles in the regressive phase (Mp4; Figure 4d), with large tubules and with abundant debris within the lumens due to a flaking germinative epithelium; spermatozoa were still present in some tubules. In the lumen of the epididymis, there was cell debris and flaked cells (Figure 5c). 

The diameter of the seminiferous tubules varied according to breeding status and was also related to the characteristics of the germinal epithelium described above (Table 4). 

Integration of male reproductive data allowed us to assign individuals to four reproductive phases, which included immature, pubertal, spermatogenesis, and regressive. According to the assigned reproductive status, male squirrels were seasonally distributed, as shown in Table 5.

## 4. Discussion

Invasive alien grey squirrels have spread since they were accidentally released in a wildlife park near Perugia in the early 2000s [16]. At the beginning of the LIFE BIO/IT204 U-SAVEREDS campaign, the estimated number of animals sighted in 2015 were 1510 grey squirrels and 112 red squirrels across an area of 13.5 km^2^ close to Perugia. More than 40%, i.e., 627 out of the 1510 grey squirrels, were sighted across 2.8 km^2^ [17]. To the best of our knowledge, this study offers the first analysis of the reproductive performance of the grey squirrels that have invaded the region of Umbria in central Italy.

The morphology and dimensions of the female reproductive organs varied according to their cyclic changes and breeding period. In particular, although no statistical differences were observed in ovary weight, uterine weights were low during the immature, follicular, and anoestrus phases and increased in weight during the luteal and pregnant phases. In addition, the morphometric parameters (length and width) of both uterine body and horns were at their highest values during pregnancy. These anatomical variations were probably due to endocrine stimulation of the genital apparatus in preparation and in support for pregnancy [24].

Data obtained from female squirrels confirm that animals in the luteal phase and pregnancy were distributed between December and March and between May and July. Accordingly, females in oestrus were observed from December to February and from April to June. Females in anoestrus were found in several months of the year, and reached the highest numbers in November, which is the month in which they are known to be sexually inactive [24,42,43]. A few females in anoestrus were also found in March, which is the month following the first period of pregnancy. Unfortunately, no female squirrels were captured from August to October, a period that is considered to be characterized by sexual inactivity and lactation by some authors [24,43,44]; thus, we can only conclude that the females of our population were inactive during this period. 

The uterine scar analysis showed that a female can produce between one and seven puppies per litter. The birth rate estimated from the uterine scar count is similar in Southeast Ohio [45] and in Northern Italy [15] and ranges from a minimum of 2−3 [21,46] to a maximum of eight [47] if the young in nests and foetuses are included. However, it is important to note that litter size is influenced by food availability during the previous winter [46,48]. Our uterine scar analysis revealed 4.5 scars/female in a year, thus indicating a larger mean litter size than those observed in their native territory of Southeast Ohio [45] and in their non-native territories in Northern Italy [15]. Our data on mean litter size ranges from 3.55 scars/female in the first litter of the year to 2.14 scars/female in the second litter of the year; in Illinois it was 2.66 scars/female in the first season and 2.44 scars/female in the second season [21]. These data obtained in native and non-native territories (Central Italy–Umbria) confirm that more uterine scars were observed in the first reproductive season, which is in contrast with Northern Italy where larger litter sizes, estimated by counting uterine scars, were observed in the second reproductive period (1.94 first period vs. 2.61 second period, total average 3.89; [15]), as also reported by other authors in North America [49] and Great Britain [22,23]. Moreover, there are more females that produce one litter compared to those that produce two, which is in contrast with the situation in Northern Italy [15]. When compared with the data obtained in its native and non-native territories, our data confirm that the Umbrian population is well adapted with regard to its reproductive output and thus potential population growth. 

Overall, our data suggest that the females within the Umbrian grey squirrel population have two mating seasons, one from December to February and the other from April to June, which is in line with the mating seasons reported for grey squirrels living in their natural habitat in North Carolina [11] or those that invaded Great Britain [24,43,44], where the two annual mating peaks are observed in March–April and June–July. Interestingly, one female in the luteal phase was captured in mid-December, while another female in late pregnancy was captured in mid-January. Considering that gestation lasts 44 days [11,24], this finding suggests that Umbrian grey squirrel females may go into oestrus earlier from December until the end of February, which covers a longer period than the oestrous cycles previously described [11,21,43,44]. A similar breeding trend was observed for precocious females living in their native habitat of North Carolina following an abundant mast crop [50]. Our data suggest that the following four periods occur throughout the year: two mating and pregnancy seasons in winter (December−February) and spring (April−June), with numerous animals in follicular (oestrus), luteal, and pregnant phases and few animals in anoestrus; another period from March to April, with immature and anoestrus animals, probably including young animals that have recently reached puberty, and lactating females following their first litter; and lastly, a period of inactivity from mid-summer until autumn, with the majority of females in anoestrus.

In male squirrels, the morphometric parameters of their testicles as well as the histological characteristics of their germinal epithelium showed considerable differences in age and breeding status. In particular, testicle size (weight, length, and width) as well as the diameters of the seminiferous tubules increased significantly during spermatogenesis. Based on histological results (morphotypes), Umbria male grey squirrels were divided into four groups in agreement with previous studies [42,51]: immature, pubertal, sexually active (spermatogenesis), and regression. However, no squirrels with recrudescing testicles were observed, which is in contrast with previous results [51]. Interestingly, the largest number of adult squirrels in spermatogenesis were observed between January and June, i.e., the same period in which all of the females in follicular and luteal phases were captured.

Although immature female grey squirrels were found throughout the year, immature males were only captured in winter, summer, and autumn. Since squirrels younger than 6−7 months are considered immature [11,35], those captured in the first months of the year were probably born in the second litter of the previous year, while males captured from June to August or later were probably born in the first or second litters of the same year. Pubertal male squirrels were observed from February to March and from November to December. Since squirrels become sexually mature at 10 to 12 months of age [11,42], we assume that pubertal squirrels captured in winter were born in the previous year, while those captured in autumn were born in the same year. 

A few animals with testicular regression were captured in February and March and only one in November; conversely, several animals were in active spermatogenesis during the same months. Previous studies have reported that North American male grey squirrels undergo a period of testicular regression and sexual inactivity in almost all months of the year [35]. Conversely, our results suggest that testicular regression only affects few animals as an individual phenomenon [26,51]. Several authors have observed that testicular regression lasted longer in years of inadequate food availability, thus suggesting that testicular regression is clearly related to food availability in the habitat [24,43,52]. In our study, squirrels with testicular regression accounted for 20.8% of the adult male population (# regression males/# spermatogenesis plus regression males) and only found in a brief period of the year. These data indicate that testicular regression is an uncommon occurrence in the Umbrian grey squirrel population, probably due to the abundance of fruit trees in this region.

These data also demonstrate that this IAS has achieved a high degree of adaptation to the local environment. Interestingly, the percentage of adult Umbrian male squirrels with testicles in the regressive phase was lower than for grey squirrels living in North America [42], 20.8% vs. 30.9%, respectively. Conversely, the percentage of young squirrels that had not reached spermatogenesis (# immature plus pubertal males/# all captured males) was much higher (52.9%) than the population that spread in the UK (32.2%; [51]). Among the adult males, the percentage of Umbrian squirrels in spermatogenesis was 10% higher than the percentage reported for the North American population [42]. Overall, these data confirm that this alien squirrel population has adapted well to its new Umbrian habitat, characterized by abundant food availability and by the absence of predators. 

By combining the reproductive data of both female and male squirrels, this study shows that the breeding of grey squirrels living in Umbria is dictated by the oestrous cycle of the females, which occurs twice per year, as previously reported for this species [42,51]. The breeding season of the Umbrian squirrels occurred in the first half of the year, especially from January to February and from April to June. From July to December, both males and females show reduced sexual activity as most females are in anoestrus, and few males are in active spermatogenesis. 

## 5. Conclusions

This study unambiguously reveals that the grey squirrel population living in Umbria has high reproductive success with an anticipated onset of the breeding season comparable to that reported for grey squirrels in native populations. Our data confirm that the Umbrian grey squirrels are well-adapted to their new environment, thus posing a considerable threat to the native red squirrel populations living in that region.

## Figures and Tables

**Table 1:** Female breeding status according to body weight and the morphometric characteristics of the genital tract.

Reproductive Phase	Body Weight (g)	Ovary Status	Ovary Weight (mg)	Ovary Length (cm)	Ovary Width (cm)	Uterus Appearance	Uterus Weight (g)	Uterine Scar	Uterine Horn Length (cm)	Uterine Horn Width (cm)	Uterine Body Length (cm)	Uterine Body Width (cm)
Immature	438.01 ^a^ ± 43.24	No graafian follicle	0.24 ^a^ ± 0.21	0.37 ^a^ ± 0.10	0.24 ^a,b^ ± 0.06	Small, ribbon-like	0.22 ^a^ ± 0.08	No	6.4 ^a^ ± 1.8	0.20 ^a^ ± 0.4	0.76 ^a,b,c^ ± 0.31	0.41 ^a,b,c^ ± 0.18
Follicular	521.54 ^a,b^ ± 55.5	Graafian follicle	0.23 ^a^ ± 0.04	0.38 ^a^ ± 0.06	0.27 ^a,b^ ± 0.05	Round, oedematous, tonic in the oestrous phase	0.82 ^a^ ± 0.49	Yes/No	9.6 ^b^ ± 3.1	0.35 ^b^ ± 0.11	1.01 ^a,b,c^ ± 0.38	0.64 ^a,b,c^ ± 0.16
Luteal	562.99 ^b^ ± 19.49	Corpus luteum	0.64 ^a^ ± 0.48	0.49 ^a^ ± 0.19	0.36 ^b.c^ ± 0.19	Round, oedematous, tonic	2.08 ^b^ ± 0.53	Yes/No	13.3 ^c^ ± 2.8	0.46 ^c^ ± 0.08	1.24 ^a,b,c^ ± 0.29	0.76 ^b^ ± 0.15
Pregnant	550.02 ^b^ ± 11.55	Corpus luteum in the first period	0.21 ^a^ ± 0.05	0.42 ^a^ ± 0.06	0.32 ^b,c^ ± 0.03	Round, oedematous, tonic, with embryonic vesicles/embryos/foetuses	3.28 ^b^ ± 1.05	No	15.5 ^c^ ± 3.9	0.51 ^c^ ± 0.13	1.45 ^b^ ± 0.33	0.98 ^b^ ± 0.40
Anoestrous	495.38 ^a,b^ ± 57.99	No graafian follicle	0.33 ^a^ ± 0.20	0.39 ^a^ ± 0.07	0.26 ^a,b^ ± 0.05	Small, ribbon-like	0.57 ^a^ ± 0.30	Yes/No	9.4 ^b^ ± 2.2	0.34 ^b^ ± 0.08	0.96 ^c^ ± 0.26	0.56 ^c^ ± 0.09

Values are indicated as mean ± standard deviation. Different superscript letters indicate significantly different values among reproductive phases. ANOVA: Body weight F_0.01; 4.35, 3.91_ = 4.03, *p* < 0.01; Ovary weight F_0.01; 4.31, 3.99_ = 2.77, *p* > 0.01; Ovary length F_0.01; 4.101, 3.20_ = 3.03, *p* > 0.01; Ovary width F_0.01; 4.91, 3.53_ = 4.54, *p* < 0.01; Uterus weight F_0.01; 4.35, 3.91_ = 32.22, *p* < 0.001; Uterine horn length F_0.01; 4.79, 3.57_ = 18.99, *p* < 0.001; Uterine horn width F_0.01; 4.79, 3.57_ = 16.42, *p* < 0.001; Uterine body length F_0.01; 4.33, 3.95_ = 4.04, *p* < 0.01; Uterine body width F_0.01; 4.33, 3.95_ = 6.60, *p* < 0.001.

**Table 2:** Seasonal distribution of female squirrels according to their reproductive phases.

	Reproductive Phase					
Catch period	Immature	Follicular	Luteal	Pregnant	Anoestrus	Total
Winter 15/12–15/03	1	4	6	7	4	21
Spring 15/03–15/06	1	9	6	2	6	24
Summer 15/06–15/09	1	-	-	1	-	2
Autumn 15/09–15/12	2	-	-	-	14	16
Total	5 (7.81%)	13 (20.31%)	12 (18.75%)	10 (15.65%)	24 (37.5%)	63

**Table 3:** Litter size estimated according to the uterine scar analysis.

Litter Presence	No. of Females	Litter Size		
None	14 (31%)	Period	Number of scars/female	Uterine scars range
One	17 (38%)	15/12–15/03	3.55 ± 1.88 *	1−7
Two	14 (31%)	16/03–15/06	2.14 ± 1.46	2−6
Total	45	Annual	4.52 ± 1.88	1−7

Litters/female values are indicated as mean ± standard deviation. * *p* < 0.01, 15/12–15/03 period vs. 16/03–15/06 period (F_0.01, 1.31, 7.56_ = 7.98).

**Table 4:** Male breeding status according to testicle morphometric characteristics and body weight.

Reproductive Phase	Body Weight (gr)	Testicle Weight (gr)	Testicle Length (cm)	Testicle Width (cm)	Epididymis Weight (gr)	ST Diameter (µm)	Testicle Position	Germinative Epithelium Morphotype	SG
Immature	399.25 ^a^ ± 70.11	0.10 ^a^ ± 0.03	0.84 ^a^ ± 0.23	0.44 ^a^ ± 0.15	0.19 ^a^ ± 0.16	79.93 ^a^ ± 18.70	ABD	Mp1	No
Pubertal	460.18 ^a,b^ ± 43.67	0.69 ^b^ ± 0.29	1.82 ^b^ ± 0.18	0.88 ^b^ ± 0.17	0.85 ^b^ ± 0.60	151.49 ^b^ ± 20.05	SCR	Mp2	No
Spermatogenesis	502.31 ^b^ ± 46.37	2.52 ^c^ ± 0.26	2.00 ^b^ ± 0.46	1.34 ^c^ ± 0.46	2.01 ^c^ ± 0.62	241.18 ^c^ ± 26.38	SCR	Mp3	Yes
Regression	515.40 ^b^ ± 36.11	1.85 ^d^ ± 0.17	2.01 ^b^ ± 0.22	1.11 ^b,c^ ± 0.16	0.36 ^a,b^ ± 0.22	166.52 ^b^ ± 55.35	SCR	Mp4	No

ABD: abdomen. SCR: scrotum. Mp: morphotype. SG: Spermatogenesis. ST: seminiferous tubules. Values are indicated as mean ± standard deviation. Different superscript letters indicate significantly different values among reproductive phases. ANOVA: Body weight F_0.01; 3.44, 4.26_ = 11.60, *p* < 0.001; Testicle weight F_0.01; 3.36, 4.38_ = 361.82, *p* < 0.001; Testicles length F_0.01; 3.43, 4.27_ = 59.99, *p* < 0.001; Testicle width F_0.01; 3.43, 4.27_ = 32.27, *p* < 0.001; Epididymis weight F_0.01; 3.38, 4.34_ = 38.01, *p* = 1.58 × 10^−11^; ST diameter, F_0.01; 3.44, 4.26_ = 95.89, *p* < 0.001.

**Table 5:** Seasonal distribution of male squirrels according to their reproductive status.

	Reproductive Phase				
	Immature	Pubertal	Spermatogenesis	Regression	Total N
Winter 15/12–15/03	4	9	10	3	26
Spring 15/03–15/06			5	1	6
Summer 15/06–15/09	5		2		7
Autumn 15/09–15/12	7	2	2	1	12
Total	16 (31.37%)	11 (21.57%)	19 (37.26%)	5 (9.80%)	51
